# No evidence for stratified exercise therapy being cost-effective compared to usual exercise therapy in patients with knee osteoarthritis: Economic evaluation alongside cluster randomized controlled trial

**DOI:** 10.1016/j.bjpt.2022.100469

**Published:** 2022-12-21

**Authors:** Jesper Knoop, Jonas Esser, Joost Dekker, J. Willemijn de Joode, Raymond W.J.G. Ostelo, Johanna M. van Dongen

**Affiliations:** a Department of Health Sciences, Amsterdam Movement Sciences, Vrije Universiteit Amsterdam, De Boelelaan 1105, Amsterdam, HV 1081, the Netherlands; b Department of Rehabilitation Medicine, Amsterdam UMC, Location VUmc, Amsterdam, the Netherlands; c Department of Epidemiology and Data Science, Amsterdam Movement Sciences, Amsterdam UMC, Location VUmc, Amsterdam, the Netherlands

**Keywords:** Cluster randomized controlled trial, Cost effectiveness, Dietary intervention, Economic evaluation, Exercise therapy, Knee osteoarthritis

## Abstract

**Background:**

A stratified approach to exercise therapy may yield superior clinical and economic outcomes, given the large heterogeneity of individuals with knee osteoarthritis (OA).

**Objective:**

To evaluate the cost-effectiveness during a 12-month follow-up of a model of stratified exercise therapy compared to usual exercise therapy in patients with knee OA, from a societal and healthcare perspective.

**Methods:**

An economic evaluation was conducted alongside a cluster-randomized controlled trial in patients with knee OA (*n* = 335), comparing subgroup-specific exercise therapy for a ‘high muscle strength subgroup’, ‘low muscle strength subgroup’, and ‘obesity subgroup’ supplemented by a dietary intervention for the ‘obesity subgroup’ (experimental group), with usual (‘non-stratified’) exercise therapy (control group). Clinical outcomes included quality-adjusted life years – QALYs (EuroQol-5D-5 L), knee pain (Numerical Rating Scale) and physical functioning (Knee Injury and Osteoarthritis Outcome Score in daily living). Costs were measured by self-reported questionnaires at 3, 6, 9 and 12-month follow-up. Missing data were imputed using multiple imputation. Data were analyzed through linear regression. Bootstrapping techniques were applied to estimate statistical uncertainty.

**Results:**

During 12-month follow-up, there were no significant between-group differences in clinical outcomes. The total societal costs of the experimental group were on average lower compared to the control group (mean [95% confidence interval]: € 405 [-1728, 918]), albeit with a high level of uncertainty. We found a negligible difference in QALYs between groups (mean [95% confidence interval]: 0.006 [-0.011, 0.023]). The probability of stratified exercise therapy being cost-effective compared to usual exercise therapy from the societal perspective was around 73%, regardless of the willingness-to-pay threshold. However, this probability decreased substantially to 50% (willingness-to-pay threshold of €20.000/QALY) when using the healthcare perspective. Similar results were found for knee pain and physical functioning.

**Conclusions:**

We found no clear evidence that stratified exercise therapy is likely to be cost-effective compared to usual exercise therapy in patients with knee OA. However, results should be interpreted with caution as the study power was lower than intended, due to the Coronavirus disease (COVID-19) pandemic.

## Highlights

• Current evidence showed that exercise therapy is a cost-effective treatment in knee OA.
• Our stratified approach of exercise therapy did not appear to be cost-effective compared to usual exercise therapy.
• Policy-makers should decide whether stratified exercise therapy can be implemented.

## Introduction

Knee osteoarthritis (OA) is one of the most common chronic health conditions (prevalence 3.8%) and one of the most disabling diseases among adults.^1^ Moreover, it results in an enormous economic burden. Medical costs – mostly from total knee replacements (TKRs) - are estimated at 460 billion dollar each year worldwide,^2^ while costs related to reduced work ability have been estimated to be 4 times higher than the medical costs in OA.^3^ Due to population aging and the rise of obesity, the already high prevalence of knee OA will keep increasing substantially in the upcoming decades^1^; therefore strategies are needed to limit this growing economic burden.

Exercise therapy is recommended as a first-step treatment for knee OA, next to pain medication and diet, with TKR to be considered only after these conservative treatments fail.^4^ There is strong evidence for the effectiveness of exercise therapy on knee pain (high quality evidence) and physical functioning (moderate quality evidence), compared to no exercise therapy.^5^, ^6^, ^7^, ^8^ This effect of exercise therapy is larger than or at least similar to any other conservative treatment^5^^,^^9^ and have not only been found in mild OA, but also in severe, end-stage OA.^10^^,^^11^ Moreover, exercise therapy is a low-cost^5^ and cost saving^12^ treatment option, with numerous studies^13^, ^14^, ^15^, ^16^, ^17^, ^18^, ^19^, ^20^, ^21^, ^22^ demonstrating its cost-effectiveness compared to no exercise therapy. Despite this, the average effect size is only modest.^5^, ^6^, ^7^, ^8^ This non-optimal outcome may be attributed to the ‘one-size-fits-all’ approach of current exercise regimens. Given the large heterogeneity of individuals with knee OA,^23^ a stratified approach to exercise therapy may yield superior clinical and economic outcomes.^24^

We recently conducted a randomized controlled trial (RCT) in knee OA to assess the clinical and cost-effectiveness of a stratified approach of exercise therapy – distinguishing a ‘high muscle strength subgroup’ (HMS), ‘low muscle strength subgroup’ (LMS) and an ‘obesity subgroup’ (OS) - compared to usual exercise therapy. As reported elsewhere,^21^ we found no added value in terms of clinical outcomes of this stratified approach compared to usual exercise therapy.^25^ This result aligns with that of multiple other trials in musculoskeletal patient groups, where stratified physical therapy appeared to have no added value,^26^, ^27^, ^28^, ^29^, ^30^, ^31^ with only one trial demonstrating a (minimal) effect of stratified physical therapy over usual care in low back pain.^32^ It could be argued, however, that despite having no added value in terms of clinical outcomes, stratified exercise therapy approaches such as ours are potentially more efficient (i.e., similar effect in less consultations).

Therefore, the aim of the current study is to evaluate the cost-effectiveness of stratified exercise therapy compared to usual exercise therapy, from the societal and healthcare perspectives.

## Methods

### Design

This economic evaluation was conducted alongside a 12-month pragmatic, parallel, 2-group cluster-randomized controlled trial (cRCT) in primary care: the OCTOPuS trial (Optimization of exerCise Therapy in patients with knee Osteoarthritis in a Primary care Setting).^25^^,^^33^ This trial was reported according to the CONSORT 2010 checklist for RCTs and the Consolidated Health Economic Evaluation Reporting Standards.

A total of 61 physical therapy practices (clusters) with 137 physical therapists were randomly allocated (1:2 ratio) to the experimental (23 practices with 54 physical therapists) or control arm (38 practices with 83 physical therapists), by using a web-based randomization program, with random sequence generation and concealment of randomization guaranteed. An independent researcher who was blinded to treatment allocation performed the randomization of physical therapy practices and supervised the blinded, primary (effectiveness) analyses. In addition, 21 dieticians (each of them linked to an experimental arm physical therapy practice) were recruited to provide the diet intervention in patients from the ‘obesity subgroup’. More details are reported elsewhere.^25^^,^^33^

### Ethics approval and consent to participate

This study was approved by the Medical Ethical Committee of the VU University Medical centre (2018.563). We conducted this study in agreement with the declaration of Helsinki (2013), in accordance with the Dutch Medical Research Involving Human Subjects Act (WMO), and the General Data Protection Regulation (in Dutch: Algemene Verordening Gegevensbescherming, AVG). We obtained written informed consent from each participant, after the information letter was provided. The researchers made sure that the participants were given complete, adequate, written and oral information regarding the nature, aims, possible risks and benefits of the study. We explained to the participants that they were free to interrupt their participation in the study at any moment without any consequence, and that they were able to receive a digital copy of their personal data. Participants received a copy of the information letter and informed consent form. An independent clinician/epidemiologist was appointed to provide participants the opportunity to ask questions about the study. The research protocol and statistical analysis plan are accessible at the Netherlands National Trial Register (https://www.trialregister.nl/trial/7463) and Open Science Framework (https://osf.io/x3p94/).

### Participants

Participants were recruited by participating physical therapists in primary care. The following inclusion criteria applied: presence of knee pain with duration ≥3 months, severity of knee pain ≥2/10 on the Numeric Rating Scale (NRS),^34^ and clinical knee OA diagnosis.^35^ Participants were excluded when meeting one of the following exclusion criteria: age<40 or >85 years, severity of knee pain ≥9/10 on the NRS, presence of physical or mental comorbidity severely affecting daily life and contraindicating exercise therapy, suspicion of chronic widespread pain, (planned) TKR, other reasons for knee pain, having received physical therapy or intra-articular injections for knee pain in the past 6 months or insufficient Dutch language comprehension.

### Study procedures

Physical therapists screened patients for eligibility at their first consultation. After providing informed consent, eligible patients were included and asked to complete questionnaires on clinical outcomes and costs at baseline (T0), 3-month follow-up (T3), 6-month follow-up (T6), and 12-month follow-up (T12), in addition to a questionnaire at 9-month follow-up (T9) for costs only, to avoid recall bias.^36^ Physical therapists and dieticians registered treatment fidelity parameters for each session. Patients from both study arms were allowed to receive any health care during the study period, which was monitored in the follow-up questionnaires.

### Interventions

#### Experimental intervention

**Fig. 1:**
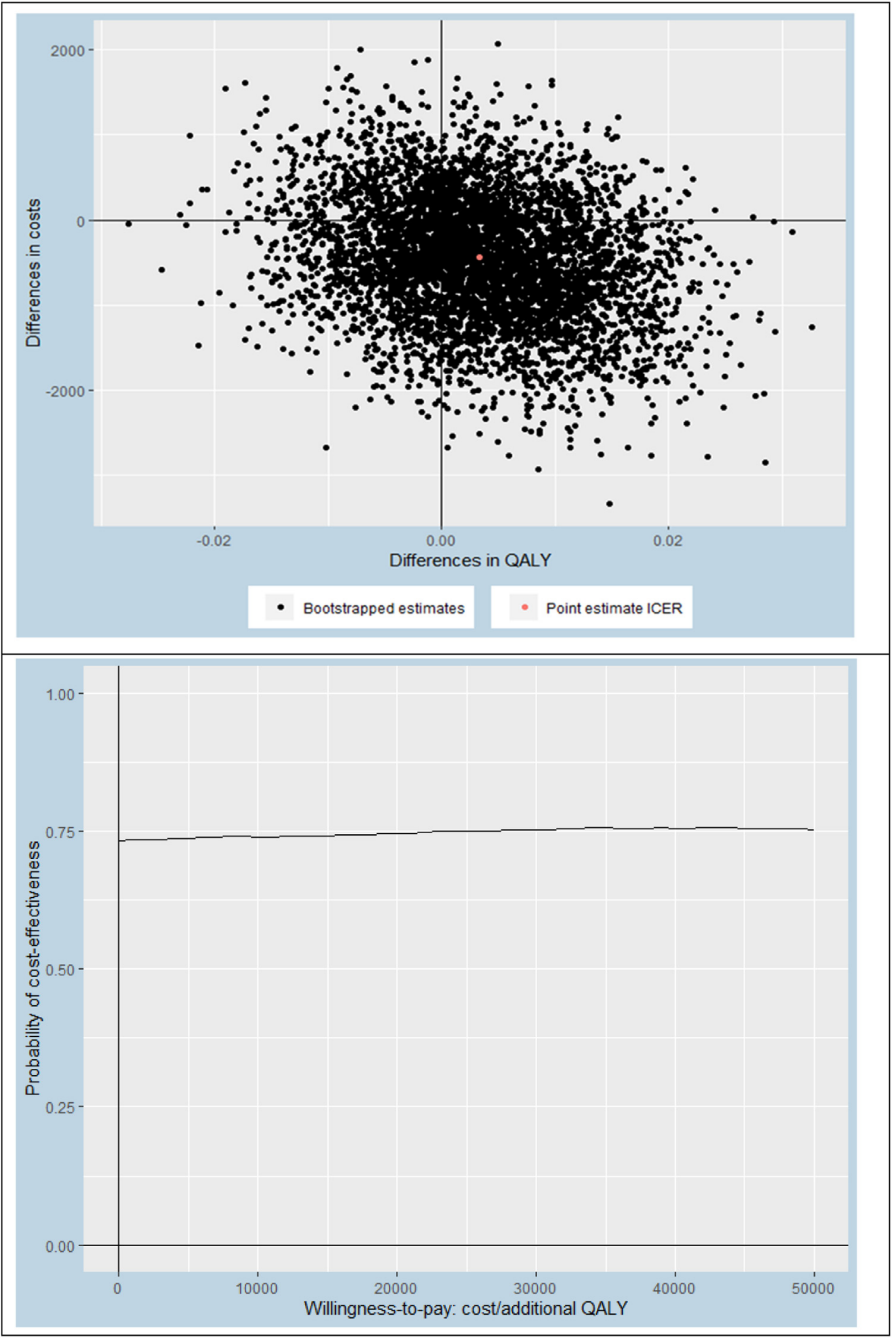
Cost-effectiveness plane (a) and cost-effectiveness acceptability curve (b), for outcome measure QALYs using a societal perspective.

Physical therapists were trained to provide the model of stratified exercise therapy and make:

(1) subgroup allocation into the HMS, LMS, or OS groups through a simple stratification algorithm with only two variables (body mass index [BMI] and upper leg muscle strength [30-seconds chair stand test]) (see Supplementary material – Fig. 1 and previous studies^25^^,^^33^).
(2) subgroup-specific, protocolized exercise therapy interventions (see Supplementary material - Table 1 and previous studies^25^^,^^33^).

**Table 1:** Baseline characteristics in OCTOPUS-trial.

	**Experimental arm (*n* = 151)**	**Control arm (*n* = 177)**
Age (years), mean ± SD	66 ± 9	64 ± 9
Sex (female), number (%)	95 (63)	114 (64)
Body mass index, kg/m^2^, mean ± SD	27.1 ± 4.0	28.6 ± 4.9
Duration of knee symptoms (years), mean ± SD	9 ± 10	7 ± 8
Using pain medication (yes), number (%)	63 (42)	86 (49)
Comorbidity affecting daily life (yes), number (%)	32 (21)	31 (18)
Work status:		
employed, number (%)	54 (36)	75 (42)
retired, number (%)	79 (53)	85 (48)
not employed, number (%)	16 (11)	16 (9)
Allocation to subgroup:		
‘high muscle strength subgroup’, number (%)	63 (42)	65 (37)*
‘low muscle strength subgroup’, number (%)	54 (35)	53 (30)*
‘obesity subgroup’, number (%)	34 (23)	53 (30)*
unknown	n/a	6 (3)*

⁎ control arm was not aware and did not use stratification algorithm for subgroup allocation.

Dieticians were instructed to deliver a dietary intervention aiming at 10% weight loss in 12 months, according to their clinical guideline to patients in the ‘obesity subgroup’ (see Supplementary material – Table 1 and previous studies^25^^,^^33^).

#### Control intervention

Physical therapists were instructed to provide their usual care according to the Dutch physical therapy guideline (i.e., standard, ‘non-stratified’ exercise therapy accompanied by patient education).

In the Netherlands, a maximum of 12 physical therapy sessions and a maximum of 180 min of dietician consultations per year are reimbursed from the ‘basic’ health insurance package for people with knee OA, from which patients are obliged to pay the first €385 themselves. In addition to the ‘basic’ health insurance package, people opt for supplementary health insurance to cover healthcare cost that are not (fully) covered by the basic health insurance package.

### Outcome measures

i. Quality-Adjusted Life Years (QALYs), assessed by the EuroQol-5D-5 L (EQ-5D-5 L; 5 items), in which the patient can self-rate their level of severity on five domains (mobility, self-care, daily activities, pain/discomfort, and anxiety/depression).^37^ The EQ-5D-5 L health states were converted into utility scores anchored at 0 (‘death’) to 1 (‘full health’) using the Dutch tariff^38^ after which QALYs were calculated using the Area Under the Curve Approach.
ii. Average knee pain severity during walking in the past week, assessed by a NRS (score 0=no pain; 10=worst pain imaginable).^34^
iii. Physical functioning, assessed by the subscale function in daily living (ADL) of the Dutch translation of the Knee Injury and Osteoarthritis Outcome Score (KOOS) questionnaire (score 0=maximal problems; 100=no problem).^39^^,^^40^
iv. Costs, valued in accordance with the Dutch Manual of Costing,^35^ expressed in Euros 2020, and subdivided into the following categories as described by Miyamoto et al.^41^:
  a. health care utilization:
    i. intervention-related costs (i.e., costs from the physical therapy sessions (valued using the Dutch tariff of €35,69 for physical therapists or €41,00/hour for manual therapists), supplemented for the ‘obesity subgroup’ of the experimental arm by the costs of the dietician sessions (valued using the Dutch tariff of €32,09/hour).
    ii. other medical costs (valued using Dutch tariffs and, if these were unavailable, prices of professional organizations) further subdivided into:
      1. primary health care, other than the (experimental or control) intervention (e.g., general practitioner, acupuncturist);
      2. secondary health care (e.g., hospital, rehabilitation center);
      3. (prescribed and over-the-counter) medication (for knee OA only);
  b. costs related to reduced work ability, with costs valued using Dutch sex-specific price weights^36^:
    i. absenteeism costs (i.e., costs of being absent from work), valued in accordance with the friction cost approach (friction period: 12 weeks)^38^^,^^41^;
    ii. presenteeism costs (i.e., costs of being less productive while being at work, based on the participant's work ability, assessed by the Work Ability Index-Single Item Scale (WAS) on a 0–10 scale^42^);
    iii. unpaid productivity costs (i.e., volunteer work, and domestic and educational activities that the participant was not able to perform), valued using a recommended Dutch shadow price of €15,14/hour^36^;
  c. informal care (i.e., care by family, friends, or other volunteers that was provided to the participant), valued using a recommended Dutch shadow price of €15,14/hour.^36^
  d. sport costs (e.g. sport shoes, fees for fitness center).

Discounting of costs was not necessary due to the 12-month follow-up period of the trial.

### Sample size

The sample size was based on the primary outcome measure of this trial (0–10 NRS knee pain). That is, based on an expected between-group difference of 0.5 on the 0–10 NRS pain scale, an estimated standard deviation (SD) of 1.4, α=0.05 (2-sided testing), power=90%, design effect of 1.05 and a 15% drop-out rate, 408 participants (204 per group) were desired.

### Statistical analysis

This economic evaluation was performed from a societal and a healthcare perspective, using a 12-month follow-up period. All participants included in the study were analyzed. Missing data were handled using Multivariate Imputation by Chained Equations (MICE) and the number of imputed datasets was determined using the loss of efficiency approach, where a loss of efficiency of <5% was deemed appropriate.^43^ Prior to multiple imputation of the data, we assessed which baseline variables differed between groups and/or were predictive of patients having missing data on one or more cost and/or effect items. These analyses indicated that BMI was the only relevant variable differing between groups. Therefore, BMI was added to the imputation model, next to all available cost and effect measure values. Pooled estimates were calculated using Rubin's rules.^41^ The average group differences in costs and effects were estimated through linear regression using identical independent variables in both equations (i.e., treatment arm and baseline values of the outcome variable, cost measure, work status, sex, age, duration of knee symptoms, upper leg muscle strength, and BMI). This is mathematically equivalent to Seemingly Unrelated Regression.^44^ Confidence intervals of the model parameters were estimated through nonparametric bootstrapping and the bias-corrected and accelerated bootstrap (BCa) method.^45^ Incremental cost-effectiveness ratios (ICERs) were estimated by dividing the differences in costs by the differences in effects. Uncertainty surrounding the ICERs were graphically illustrated by plotting bootstrapped cost-effectiveness pairs on cost-effectiveness planes. Also, cost-effectiveness acceptability curves were constructed to provide an indication of the probability of stratified exercise therapy being cost-effective compared with usual exercise therapy at different values of willingness-to-pay (ranging between €0 to €50,000 per QALY).^46^

In addition to the primary cost-effectiveness analysis, the following 5 a priori specified sensitivity analyses (SA) were performed. In SA1, the healthcare perspective was applied, meaning that only costs accruing to the formal Dutch healthcare system were included in the analyses. In SA2, the Human Capital Approach^41^ was used instead of the Friction Cost Approach for estimating absenteeism costs. In SA3, only data of patients with complete cost values at all measurement points were included. In SA4, the main analysis was performed with data from the per-protocol analysis (excluding minor and/or major protocol violators; see Supplementary material – Table 2). In SA5, the main analysis was performed, but stratified per subgroup (i.e., HMS, LMS and OS). R version 4.0.3 was used for the cost-effectiveness analysis. IBM SPSS statistics 27 was used for description of baseline characteristics.

**Table 2:** Clinical outcomes and knee-related health care utilization in OCTOPUS-trial.

	**Experimental arm (*n*** **=** **151)**	**Control arm (*n*** **=** **177)**
Clinical outcomes*	**mean (95% CI)**	**mean (95% CI)**
QALYs (EQ-5D, 0–1)		
Baseline	0.778 (0.758, 0.799)	0.765 (0.743, 0.787)
3 months follow-up	0.799 (0.774, 0.823)	0.785 (0.758, 0.812)
12 months follow-up	0.887 (0.872, 0.902)	0.877 (0.862, 0.891)
Knee pain (NRS, 0–10)		
Baseline	5.1 (4.9, 5.3)	5.3 (5.2, 5.4)
3 months follow-up	4.1 (3.9, 4.3)	4.1 (3.9, 4.3)
12 months follow-up	3.7 (3.5, 3.9)	3.9 (3.7, 4.1)
Physical function (KOOS, 0–100)		
Baseline	67.5 (66.2, 68.9)	65.2 (64.0, 66.4)
3 months follow-up	73.9 (72.6, 75.2)	72.2 (71.0, 73.5)
12 months follow-up	76.5 (75.0, 78.1)	76.6 (75.3, 77.9)
**Knee OA-related health care utilization**		
Study intervention**	**mean ± SD**	**mean ± SD**
Physical therapy sessions, mean ± SD	8.4 ± 4.7	9.6 ± 4.8
Dietary sessions, mean ± SD***	3.2 ± 1.8	n/a
Other****	**n (%)**	**n (%)**
Total knee arthroplasty, n (%)	2 (1)	2 (1)
Knee joint injections, n (%)	2 (1)	4 (2)

SD = standard deviation.

⁎ based on data from patient-reported questionnaires after multiple imputation;.

⁎⁎ based on registration data from participating physical therapists and dieticians collected during study, without multiple imputation.

⁎⁎⁎ only data from participants from ‘obesity subgroup’ in experimental arm (*n* = 34).

⁎⁎⁎⁎ based on data from participants and/or therapists, without multiple imputation.

## Results

### Participants

In total, 335 patients with knee OA were included in our trial: 153 in the experimental arm and 182 in the control arm (see Supplementary material – Fig. 2 and previous studies^25^^,^^33^). We included patients between January 2019 and May 2020, and stopped inclusion at 335 participants (instead of the intended 408) due to Coronavirus disease (COVID-19) lock-down that obstructed any further inclusion for a substantial period. Baseline and 3-month follow-up data could be collected from 328 patients (98%), who were therefore included in the intention-to-treat analyses. There were no substantial differences at baseline across the study arms, except for BMI (Table 1).

### Effects and health care utilization

No significant between-group differences in clinical outcomes were found, neither in the total sample (Table 2), nor when analyzed separately for the HMS, LMS, or OS.^25^ The average number of registered physical therapy sessions only slightly differed between treatment arms: 8.4 ± 4.7 sessions in experimental arm vs 9.6 ± 4.8 sessions in control arm. The ‘obesity subgroup’ in the experimental arm additionally received on average 3.2 ± 1.8 sessions from a dietician (Table 2).

### Costs

As shown in Table 3, only small between-group differences in health care costs were found, with the intervention costs being slightly lower in the experimental group and the other primary and secondary health care costs being slightly higher, compared to the control group. All work-related cost estimates were lower in the experimental group compared with the control group. Overall, mean adjusted total cost difference were €−405 (95% confidence interval [CI]: −1728, 918) from a societal perspective (i.e., less costs in experimental group) and €30 (95% CI: −156, 216)) from a healthcare perspective (i.e., less costs in control group).

**Table 3:** Costs per arm and cost differences across arms.

Costs during 12-month follow-up period*	**Experimental group (*n*** **=** **151), mean costs in € (95% CI)**	**Control group (*n*** **=** **177), mean costs in € (95% CI)**	**Crude mean cost difference in € (95% CI)**	Adjusted* mean cost difference in € (95% CI)
Intervention-related	370 (315, 425)	466 (409, 523)	−96 (−174, −18)	−99 (−179, −19)
Primary health care (other than intervention)⁎⁎	263 (200, 326)	204 (155, 253)	59 (−19, 137)	64 (−3, 131)
Secondary health care⁎⁎⁎	430 (232, 628)	362 (213, 511)	68 (−181, 317)	65 (−192, 322)
Medication⁎⁎⁎⁎	1 (1, 1)	1 (1, 1)	0 (−1, 11)	0 (0, 0)
Presenteeism	4219	5193	−974	−106
	(3133, 5305)	(4029, 6357)	(−2573, 625)	(−1023, 811)
Absenteeism	395 (123, 667)	665 (320, 1010)	−270 (−709, 169)	−224 (−602, 154)
Unpaid productivity	837	1022	−185	−121
	(439, 1235)	(657, 1387)	(−726, 356)	(−540, 298)
Informal care	511 (305, 717)	504 (286, 722)	7 (−293, 307)	61 (−194, 316)
Sport	196 (149, 243)	254 (197, 311)	−58 (−132, 16)	−62 (−133, 9)
**Total**	7222	8671	−1449	−405
	(5793, 8651)	(7154, 10,188)	(−4395, 1497)	(−1728, 918)

CI = confidence interval.

⁎ adjusted for treatment arm, baseline costs, QALY, work status, sex, age, duration of knee symptoms, upper leg muscle strength, and BMI.

⁎⁎ primary health care, other than the (experimental or control) intervention (e.g., general practitioner, acupuncturist).

⁎⁎⁎ secondary health care (e.g., hospital, rehabilitation center).

⁎⁎⁎⁎ (prescribed and over-the-counter) medication (for knee OA only).

### Cost-effectiveness

From a societal perspective (our main analysis), the ICER for QALYs was €−83,547/QALY, based on an adjusted mean cost difference of €−426 and an adjusted mean effect difference of 0.006 (Table 4). This ICER indicates that 1 QALY gained by stratified exercise therapy was on average associated with a cost saving of €−83,547 compared to usual exercise therapy. Hence, stratified exercise therapy was superior to usual exercise therapy. It should be noted that this ICER is relatively large due to the very small difference in QALYs (i.e., 0.006). The probability of stratified exercise therapy being cost-effective compared to usual exercise therapy was roughly 73% (regardless of the willingness-to-pay threshold; Fig. 1). When using a healthcare perspective (SA1), this probability was much lower (i.e., 38%, 50%, and 55% at willingness-to-pay thresholds of €0, €20,000, and €50,000/QALY, respectively) (see Supplementary material - Fig. 3). Comparable results were found for knee pain and physical functioning, namely very small differences in effect with slightly less costs in the experimental group from the societal perspective (main analysis) which vanished when using the healthcare perspective (SA1).

**Table 4:** Cost-effectiveness analysis.

**Analysis**	**Sample size**		**Outcome**	**∆Costs (95% CI)**	**∆Effects (95% CI)**	**ICER**	**Distribution CE-plane (%)**			
	**EXP**	**CON**		**€**	**Points**	**€/point**	**NE^a^**	**SE^b^**	**SW^c^**	**NW^d^**
**Main analysis—imputed dataset**	151	177	QALYs (range: 0–1)	−426 (−1781, 914)	0.006 (−0.011, 0.023)	−83,547	15	57	17	10
	151	177	NRS pain (range: 0–10*)	−436 (−1760, 915)	−0.07 (−0.45, 0.29)	5831	8	28	46	18
	151	177	KOOS-ADL (range: 0–100)	−417 (−1764, 893)	−0.07 (−2.79, 2.49)	5759	12	37	35	16
**SA1—health care perspective**	151	177	QALYs (range: 0–1)	50 (−217- 381)	0.006 (−0.011, 0.023)	23,098	34	26	13	27
	151	177	NRS pain (range: 0–10*)	54 (−214, 384)	−0.09 (−0.46, 0.28)	−635	20	13	25	41
	151	177	KOOS-ADL (range: 0–100)	51 (−215, 380)	−0.16 (−2.86, 2.43)	−319	27	19	20	34
**SA2—human capital approach**	151	177	QALYs (range: 0–1)	−464 (−1841, 850)	0.006 (−0.011, 0.023)	−138,995	14	51	23	12
	151	177	NRS pain (range: 0–10*)	−467 (−1829, 869)	−0.07 (−0.45, 0.29)	6244	8	28	46	17
	151	177	KOOS-ADL (range: 0–100)	−447 (−1817, 866)	−0.07 (−2.79, 2.49)	6178	11	38	36	15
**SA3—complete cases for cost outcomes only**	107	136	QALYs (range: 0–1)	239 (−1246, 1717)	0.000 (−0.017, 0.019)	−46,3329	23	22	16	39
	107	136	NRS pain (range: 0–10*)	323 (−1143, 1796)	−0.11 (−0.53, 0.31)	−2952	18	12	21	49
	107	136	KOOS-ADL (range: 0–100)	320 (−1103, 1789)	0.33 (−2.59, 3.61)	983	34	21	11	33
**SA4 – per protocol cases only (excluding major protocol violators)**	111	162	QALYs (range: 0–1)	−273 (−1654, 1238)	0.006 (−0.013, 0.023)	−56,660	21	46	20	13
	111	162	NRS pain (range: 0–10*)	−234 (−1603, 1298)	−0.06 (−0.47, 0.37)	3895	14	27	36	23
	111	162	KOOS-ADL (range: 0–100)	−254 (−1628, 1264)	−0.35 (−3.34, 2.56)	733	13	29	34	23
**SA5a– main analysis in ‘high muscle strength subgroup’ only**	63	65	QALYs (range: 0–1)	−536 (−2882, 1723)	0.005 (−0.018, 0.028)	−13,109	17	42	25	16
	63	65	NRS pain (range: 0–10*)	−492 (−2911, 1898)	−0.32 (−0.89, 0.29)	1516	4	10	56	30
	63	65	KOOS-ADL (range: 0–100)	−296 (−2659, 1997)	−1.11 (−4.40, 2.39)	267	7	18	43	32
**SA5b– main analysis in ‘low muscle strength subgroup’ only**	54	53	QALYs (range: 0–1)	−1320 (−3285, 318)	0.015 (−0.011, 0.045)	−112,457	5	65	26	4
	54	53	NRS pain (range: 0–10*)	−1199 (−3127, 531)	0.53 (−0.15, 1.21)	−2244	11	79	10	1
	54	53	KOOS-ADL (range: 0–100)	−1214 (−3173, 488)	2.85 (−1.9, 8.3)	−426	9	71	17	3
**SA5c– main analysis in ‘obesity subgroup’ only**	34	53	QALYs (range: 0–1)	386 (−2755, 4141)	0.000 (−0.046, 0.045)	86,301	26	33	14	27
	34	53	NRS pain (range: 0–10*)	238 (−2907, 4069)	−0.09 (−0.93, 0.68)	−2,5723	19	25	26	30
	34	53	KOOS-ADL (range: 0–100)	78 (−3104, 3878)	−1.27 (−7.69, 4.65)	−62	13	25	28	33

CI, confidence interval; C, costs; CE-plane, cost-effectiveness plane; E, effects; ICER, incremental cost-effectiveness ratio; SA, sensitivity analysis.

⁎ 0–10 scale for NRS pain transformed so that higher score means better (less pain) instead of worse outcome, similar as for the other outcome measures, to facilitate interpretation.

Also the human capital approach (SA2) and per-protocol analyses (SA4) showed very similar results as the main analysis, while the results from the complete case analysis (SA3) were slightly more in favor of the control arm, but again with much uncertainty. The stratified analyses for each of the 3 subgroups of the OCTOPuS algorithm separately (SA5) indicated that – compared to the 73% likelihood of stratified exercise therapy being cost-effective in the total group – this likelihood is much larger in the LMS (95%) and much lower in the OS (37%).

## Discussion

The current study showed no clear evidence that stratified exercise therapy is likely to be a cost-effective option compared to usual exercise therapy in patients with knee OA. As we did not demonstrate superiority for clinical effectiveness either,^25^ we rejected our hypothesis that this new intervention has added value over usual care. However, results should be interpreted with caution as the study power was lower than intended, due to the COVID-19 pandemic. Hence, it is up to policy-makers whether they perceive the likelihood of stratified exercise therapy possibly generating societal cost savings while maintaining clinical effects warrants implementation of this intervention in clinical practice.

The present study indicates that – although cautious interpretation is warranted – the stratified exercise therapy approach could potentially result in (particularly work-related) cost-savings, with similar clinical effects compared to usual exercise therapy. Results from separate analyses in each of the 3 subgroups of the stratified model also suggest that cost savings are most likely to occur in the LMS and to a lesser extent in the HMS, while being least likely in the OS. However, we cannot draw firm conclusions from our study because all cost difference estimates were surrounded by very high levels of uncertainty (partly due to insufficient power) and cost differences disappeared when focusing only at the healthcare perspective. Decision-makers (as well as health care professionals and patients) should therefore balance the uncertain cost savings (in work-related costs) and the lack of superior clinical effects by the stratified exercise therapy on the one hand against the effort that comes with implementing the new treatment. In this perspective, the barriers for applying the new treatment (especially for the OS) as reported elsewhere^47^ should also be acknowledged.

Our inconclusive result seems to be consistent with the scarce and conflicting evidence regarding the cost-effectiveness of stratified approaches of physical therapy, with two trials^32^^,^^48^ concluding that stratified care can be cost-effective, three trials^27^^,^^28^^,^^30^^,^^31^ concluding that stratified care is unlikely to be a cost-effective option, and a last study^49^ concluding that it might be cost-effective for only one out of three subgroups, compared to usual physical therapy. These conflicting results may be indicative for stratified care having only negligible added value to current physical therapy at best, but differences across studies in country, setting, patients groups, and stratification tool could play a role as well. Although physical therapy is being considered potentially cost-saving in musculoskeletal primary care,^24^^,^^41^ it remains therefore unclear whether stratified approaches can lead to even larger cost-savings. Future studies, including two currently ongoing studies,^50^^,^^51^ should make clear whether stratified approaches of physical therapy can have added value in musculoskeletal health.

We would like to highlight two potential reasons for the unexpected finding of stratified exercise therapy not resulting in healthcare costs savings. First, we a priori expected that our stratified approach would not only lead to superior clinical effects, but also in substantial cost savings due to less physical therapy sessions (especially in HMS), other healthcare utilization and work absenteeism. However, with regards to the physical therapy sessions, we found only a very small difference in number of sessions of 1.2 (8.4 vs 9.6) between our study arms. This can be attributed to the much lower amount of sessions in the control group than what was expected, which can be considered an indication for an (even further) improvement in efficiency by Dutch physical therapists. It could also be caused by the COVID-19 pandemic, as health care sites were closed or restricted to acute situations only. Finally, also work absenteeism occurred much less than expected in both study arms. It should be noted that half of our study group was already retired (i.e., 53% in experimental and 48% in control arm) and those wo still worked showed relatively high work ability and low sick leave rate already at baseline, so there was not much room for substantial group differences in work-related costs.

A second reason for the lack of difference in healthcare costs between our study arms is the smaller than intended contrast between the interventions, as also reported in our clinical effectiveness study.^25^ Especially in the OS, the intervention had not been applied as recommended (e.g., too few sessions) and we identified several barriers for optimally providing the combined intervention of physical therapy and diet advice.^47^ The non-optimal provision of the OS-intervention might be one of the reasons that this subgroup reported on average no reductions in weight and smaller clinical outcomes than expected. Presumably, a more extended combined treatment should be provided to reach (and sustain) clinically relevant weight loss and to result in clinical and economic effects, as for example the IDEA-intervention (i.e., 18-month intervention of 3 one-hour exercise therapy sessions/week plus 50 dietary sessions^52^). While for the short term, extensive interventions combining exercise and diet (like the IDEA-intervention) would lead to more costs, it might result in lower rates of TKR and of sick leave on a longer term (which could make it a cost-effective option). Economic evaluations should therefore have follow-up periods up to 10 years to clarify this.

We should address the following study limitations. First, as with nearly every clinical trial, our study was powered on the primary effect outcome, rather than on cost-effectiveness outcomes, such as costs. This is common practice in health economics, because costs are heavily right skewed and would therefore require extremely large sample sizes, which in turn might be infeasible and/or unethical. To deal with this issue, we used estimation (e.g. by reporting the probability of cost-effectiveness) rather than hypothesis testing for interpreting our outcomes.^41^ The study's power was lower than intended due to the COVID-19 pandemic, which forced us to prematurely terminate our trial. Second, our cost estimations were based on patient-reported cost questionnaires, which has a recall bias as a downside.^36^ Third, due to the COVID-19 pandemic in the last phase of our trial, primary and especially secondary health care had been canceled and/or postponed for some periods, which may have resulted in lower health care utilization. This could have resulted in lower health care utilization in our study group, therefore our healthcare costs may not be generalizable. However, this did not affect our study findings. Fourth, we used a number of exclusion criteria to maximize the chance of a true knee OA diagnosis (e.g., age <40 years) and to minimize the risk of (rare) complications of exercise therapy (e.g., age >85 years, pain severity *≥* 9/10, severe comorbidities contraindicating exercise therapy), which reduced the external validity of our study findings. Next to these study limitations, major strengths are that the economic evaluation was conducted alongside a pragmatic RCT and performed using the most up-to-date analyses techniques.^41^^,^^43^^,^^44^^,^^46^

## Conclusion

We found no clear evidence that stratified exercise therapy is more cost-effective compared to usual exercise therapy in patients with knee OA. However, results should be interpreted with caution as the study power was lower than intended, due to the COVID-19 pandemic. Hence, it is up to policy-makers, whether they perceive the likelihood of stratified exercise therapy possibly generating societal cost savings while maintaining clinical effects, to determine if implementation of this intervention in clinical practice is warranted.

## Conflicts of interest

All authors declare that they have no competing interests.

## Appendix Supplementary materials

Image, application 1
