# Supplementary material for: No evidence for stratified exercise therapy being cost-effective compared to usual exercise therapy in patients with knee osteoarthritis: Economic evaluation alongside cluster randomized controlled trial
Source: Braz J Phys Ther. 2022 Dec 21;27(1):100469. doi: 10.1016/j.bjpt.2022.100469 (PMC9860430; doi:10.1016/j.bjpt.2022.100469)
Supplement: Supplementary file 1 [file mmc1.docx]

**Supplementary material – table 1: Subgroup-specific, protocolized interventions**

| **‘High muscle strength subgroup’** | **‘Low muscle strength subgroup’** | **‘Obesity subgroup’** |
| --- | --- | --- |
| ***Exercise therapy from physical therapist***  Number of sessions:   - 3-5 individual sessions in 12-week treatment period - 1 ‘booster’ session in post-treatment period   Content:   1. subgroup-specific education/ advice 2. home exercises | ***Exercise therapy from physical therapist***  Number of sessions:   - 8-12 individual sessions in 12-week treatment period - 1-2 ‘booster’ sessions in post-treatment period   Content:   1. subgroup-specific education/ advice 2. supervised exercise therapy, primarily targeting upper leg muscle strength 3. home exercises | ***Exercise therapy from physical therapist***  Number of sessions:   - 12-18 individual sessions in 12-week treatment period - 2-3 ‘booster’ sessions in post-treatment period   Content:   1. subgroup-specific education/ advice 2. supervised exercise therapy adapted to obesity, targeting upper leg muscle strength, aerobic capacity and weight loss 3. home exercises |
|  |  | ***Dietary intervention from dietician***  Number of sessions:   - 5-8 individual sessions, of 150 minutes in total   Content:   - advising and monitoring healthy diet and active lifestyle, aiming at ≥ 10% weight loss |
|  |  | ***Interprofessional consultation between physical therapist and dietician***   - at least one consultation after 3 to 4 weeks of treatment to agree on an approach to achieve sustainable lifestyle change |

**Supplementary material – Table 2: Definitions of major and minor protocol violations**

*Major protocol deviations are defined as one of the following:*

- For both treatment arms:
  - undergoing total knee arthroplasty between baseline and 12-months follow-up; or
  - undergoing knee injection between baseline and 12-months follow-up; or
  - undergoing sleeve gastrectomy (stomach reduction) between baseline and 12-months follow-up.
- Specifically for control arm:
  - ≤ 2 physical therapy sessions.
- Specifically for experimental arm:
  - for all three subgroups:
    - < 2 months of physical therapy treatment period (recommended: 3 months)
  - for ‘high muscle strength subgroup’ only:
    - ≤ 2 physical therapy sessions in 3-month treatment period (recommended: 3-5 sessions); or
    - absence of the recommended physical therapy interventions: patient education/advice and home exercises
  - for ‘low muscle strength subgroup’ only:
    - ≤ 6 physical therapy sessions in 3-month treatment period (recommended: 8-12 sessions); or
    - absence of the recommended physical therapy interventions: patient education/advice, supervised exercise therapy and home exercises
  - for ‘obesity subgroup’ only:
    - ≤ 10 physical therapy sessions in 3-month treatment period (recommended: 12-18 sessions); or
    - < 3 dietician sessions in 12-month treatment period (recommended: 5-8 sessions); or
    - absence of the recommended physical therapy interventions: patient education/advice, supervised exercise therapy and home exercises.

*Minor protocol deviations are defined as one of the following:*

- Only for experimental arm:
  - for ‘high muscle strength subgroup’ only:
    - ≥ 7 physical therapy sessions in 3-month treatment period (recommended: 3-5 sessions);
  - for ‘low muscle strength subgroup’ only:
    - ≥ 14 physical therapy sessions in 3-month treatment period (recommended: 8-12 sessions), or
    - no ‘booster physical therapy session’ between 3-months and 12-month follow-up (recommended: 1-2 ‘booster sessions’);
  - for ‘obesity subgroup’ only:
    - ≥ 20 physical therapy sessions in 3-month treatment period (recommended: 12-18 sessions); or
    - no ‘booster physical therapy session’ between 3-months and 12-month follow-up (recommended: 2-3 ‘booster sessions’); or
    - ≥ 10 dietician sessions in 12-month treatment period (recommended: 5-8 sessions).

**Supplementary material – Figure 1: OCTOPuS stratification algorithm**


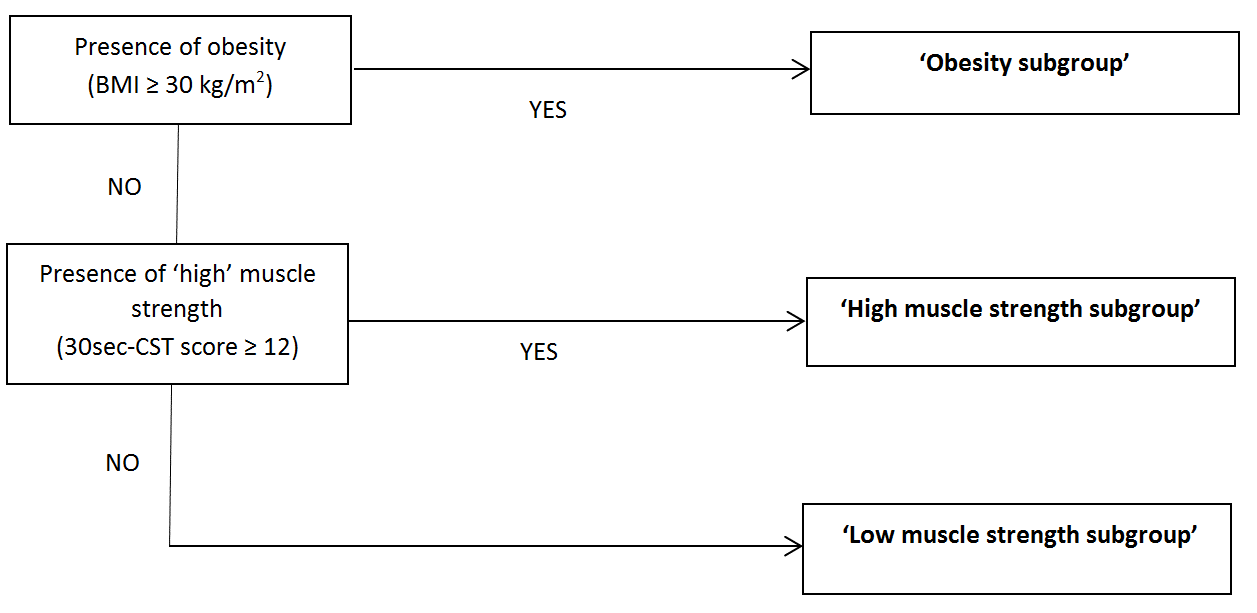


**Supplementary material – Figure 2: Flow chart OCTOPUS-study**

Excluded (n=18)

♦ Not meeting inclusion criteria (n=4)

- > 50km from research center (n=2)
- < 1 knee OA patient/month (n=2)

♦ Declined to participate (n=14)

Cluster enrollment

Recruitment of PTs (n=155)

PTs withdrawn before start trial (n=2)

♦ Stopped working at PT practice (n=2)

PTs discontinued during trial (n=2)

♦ Stopped working at PT practice (n=2)

Excluded (n=137)

♦ Not meeting inclusion criteria (n=105)

♦ Declined to participate (n=32)

Patients tested for eligibility (n=298)

Patients tested for eligibility (n=290)

Randomization of PTs on level of PT practice (n=137)

Excluded (n=116)

♦ Not meeting inclusion criteria (n=79)

♦ Declined to participate (n=37)

PTs withdrawn before start trial (n=5)

♦ Declined to participate (n=3)

♦ Stopped working at PT practice (n=2)

PTs discontinued during trial (n=7)

♦ Stopped working at PT practice (n=5)

♦ Not willing to participate anymore (n=2)

PTs in experimental group (n=54) + dieticians (n=21) for ‘obesity subgroup’

PTs in control group (n=83)

Cluster allocation

Patients assessed at:

T0 (n=177) T3 (n=165)
T6 (n=156)
T9 (n=148)
T12 (n=155)

Included and provided informed consent (n=182)

Patient enrollment

Follow-Up

Analysis

Analysed in ITT T0-T3 (n= 151)

Analysed in per-protocol T0-T3 (n=120/107):
♦ Excluding major violators (n=31)
♦ Excluding major and minor violators (n=44)

Analysed in ITT T0-T12 (n=151)

Analysed in per-protocol T0-T12 (n=111/69):
♦ Excluding major violators (n=40)
♦ Excluding major and minor violators (n=82)

Patients assessed at:

T0 (n=151) T3 (n=141)
T6 (n=130)
T9 (n=123)
T12 (n=131)

Lost to FU before T3 (n=4)
♦ Withdrawn before T0 (n=2)

♦ Withdrawn between T0-T3 (n=2)

Lost to FU after T3 (n=15)
♦ Withdrawn between T3-T12 (n=15)

‘obesity subgroup’ (n=35)

‘low muscle strength subgroup’ (n=54)

Included, provided informed consent and allocated (n=153):

Analysed in ITT T0-T3 (n=177)

Analysed in per-protocol T0-T3 (n=165/165):
♦ Excluding major violators (n=12)
♦ Excluding major and minor violators (n=12)

Analysed in ITT T0-T12 (n= 177)

Analysed in per-protocol T0-T12 (n=162):
♦ Excluding major violators (n=15)
♦ Excluding major and minor violators (n=15)

Lost to FU before T3 (n=11)
♦ Withdrawn before T0 (n=5)

♦ Withdrawn between T0-T3 (n=6)

Lost to FU after T3 (n=11)
♦ Withdrawn between T3-T12 (n=11)

**Supplementary material – Figure 3: Cost-effectiveness plane (a) and cost-effectiveness acceptability curve (b), for outcome measure QALYs using a healthcare perspective**

‘high muscle strength subgroup’ (n=64)

| 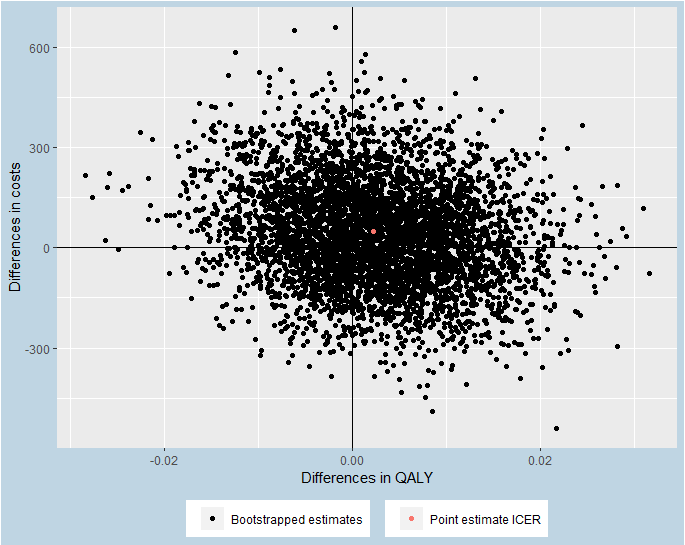 |
| --- |
| 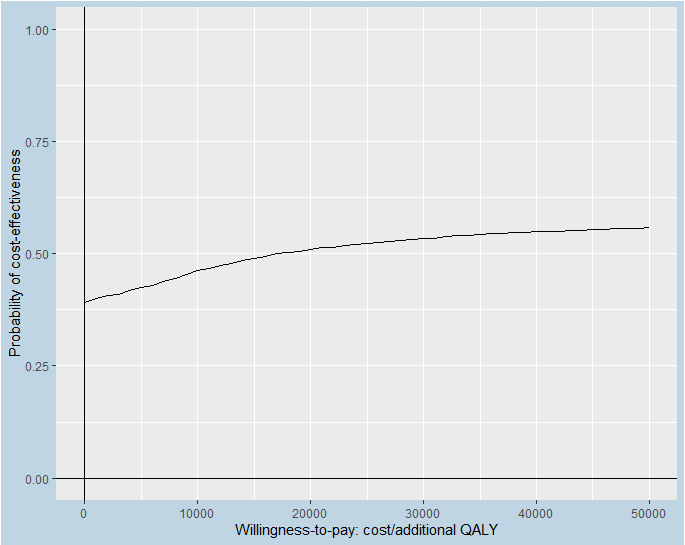 |
